# The association between acute myocardial infarction and subsequent diagnosis of breast cancer: a nationwide, population-based cohort study

**DOI:** 10.1038/s41598-024-66141-7

**Published:** 2024-07-09

**Authors:** Chia-Pin Lin, Shing-Hsien Chou, Yu-Sheng Lin, Hou-Yu Chiang, Chan-Keng Yang, Yu-Sheng Lin, Pao-Hsien Chu

**Affiliations:** 1 Division of Cardiology, Department of Internal Medicine, Chang Gung Memorial Hospital, Linkou, Taiwan; 2 School of Medicine, National Tsing Hua University, Hsinchu, Taiwan; 3 Graduate Institute of Clinical Medical Sciences, College of Medicine, Chang Gung University, Taoyuan, Taiwan; 4 Division of Cardiology, Chiayi Branch, Chiayi Chang Gung Memorial Hospital, Chiayi, Taiwan; 5 Department of Anatomy, College of Medicine, Chang Gung University, Taoyuan, Taiwan; 6 Division of Hematology-Oncology, Chang Gung Memorial Hospital, Taoyuan, Taiwan; 7 Department of Internal Medicine, Taoyuan Chang Gung Memorial Hospital, Taoyuan, Taiwan; 8 Healthcare Center, Chang Gung Memorial Hospital, No. 5, Fuxing St., Guishan Dist., Taoyuan City 333, Taiwan; 9 Institute of Stem Cell and Translational Cancer Research, Chang Gung Memorial Hospital, Chang Gung University College of Medicine, No. 5, Fuxing St., Guishan Dist., Taoyuan City 333, Taiwan

**Keywords:** Acute myocardial infarction, Newly diagnosed, Breast cancer, Cardiovascular disease, Breast cancer, Cardiology, Health care, Medical research, Oncology, Risk factors

## Abstract

Coronary artery disease (CAD) such as acute myocardial infarction (MI) share several common risk factors with cancers, and each disease may influence the prognosis of the other. Recently, acute MI was demonstrated to accelerate the outgrowth of preexisting breast cancer cells but the risk of breast cancer after MI remains unclear. This study aimed to investigate the association between acute MI and a subsequent diagnosis of breast cancer. Female patients with and without a history of acute MI were identified from nationwide databases in Taiwan. Patients with a diagnosis of cancer, MI or CAD prior to the study period were excluded. After reducing confounding through inverse probability of treatment weighting, we compared the incidence of newly diagnosed breast cancer between patients with a history of acute MI and those without. As a result, a total of 66,445 female patients were obtained, including 15,263 patients with a history of acute MI and 51,182 patients without. The incidences of breast cancer during follow-up were 1.93 (95% confidence interval [CI] 1.78–2.09) and 1.80 (95% CI 1.67–1.93) per 1,000 person-years for patients with and without a history of acute MI, respectively. The hazard ratio (HR) was 1.05 (95% CI 0.78–1.41, *P* = 0.756). In subgroup analysis, breast cancer risk was significantly associated with acute MI in patients using antidiabetic drugs (HR 1.27; 95% CI 1.02–1.58) and in low to moderate urbanization levels (HR 1.28; 95% CI 1.06–1.53). In conclusion, the risk of newly diagnosed breast cancer was not increased in patients with acute MI when compared to general population without MI or CAD.

## Introduction

Coronary artery disease (CAD) is one of the leading causes of morbidity and mortality worldwide^1–3^. Mortality after acute myocardial infarction (MI) has decreased due to advancements in pharmacotherapy and better intervention practices^4^. Noncardiac causes of morbidity after acute MI, such as cancer, are gaining recognition; however, their association is still not fully understood. Cardio-oncology is currently a new research and clinical subspecialty in the field of cardiology^5–7^. Some common cardiovascular disease risk factors, such as cigarette smoking, dyslipidemia, diabetes, and obesity, are also risk factors for cancer^8^.

Breast cancer has surpassed lung cancer as the most commonly diagnosed cancer worldwide and is currently the fourth-ranked cancer-specific cause of death. In 2020, 2.3 million new cases of breast cancer were diagnosed, resulting in 685,000 deaths^9^. The incidence of breast cancer is estimated to reach 4.4 million globally by 2070^10^. Previous studies have demonstrated that the incidence of new-onset cancer, especially tobacco-related cancers, was higher in patients with a history of MI or post-MI heart failure, but this association is less clear for other cancers^11–15^. Recently, Koelwyn et al. demonstrated that MI accelerated the outgrowth of preexisting breast cancer cells and increased cancer-specific mortality in both mice and humans via innate immune reprogramming^16^. However, the pathogenesis and potential biological mechanism linking newly developed breast cancer to CAD or MI remain unclear, and only a few subgroup studies have evaluated the risk of breast cancer after MI^11,13,14,17^.

In the present study, we investigated the effect of acute MI on the risk of newly diagnosed breast cancer during the follow-up period in over 66,000 female patients using a real-world, nationwide cohort database.

## Methods

### Data source

The data used in this study were obtained from the National Health Insurance Research Database (NHIRD) and the Longitudinal Health Insurance Database 2000 (LHID2000). The National Health Insurance (NHI) program is a single-payer, nationwide, government-operated universal health insurance program implemented from 1995 in Taiwan, which covers more than 99.6% of the population. The NHIRD is an administrative claims database, which includes both inpatient and outpatient medical information and registration files from all medical facilities contracted with the NHI administration and provides comprehensive medication, operation, procedure, cause of death, and established diagnosis records of patients^18^. The LHID2000 is an original claims database of 1 million randomized individuals sampled from the Registry for Beneficiaries of the NHIRD from the year 2000. All personal identifiers in the NHIRD and LHID2000 datasets were removed by the Bureau of NHI before the release of information to researchers to protect patient privacy. All diseases were diagnosed using *International Classification of Diseases, Ninth Revision, Clinical Modification* (*ICD-9-CM*) codes. This research was performed in accordance with the Helsinki Declaration and approved by the Ethics Committee of the Institutional Review Board of Chang Gung Memorial Hospital, Linkou, Taiwan (approval number 201800819B0). Informed consent was waived because of the retrospective nature of the study and the anonymized nature of the data.

### Study cohort and design

**Figure 1:**
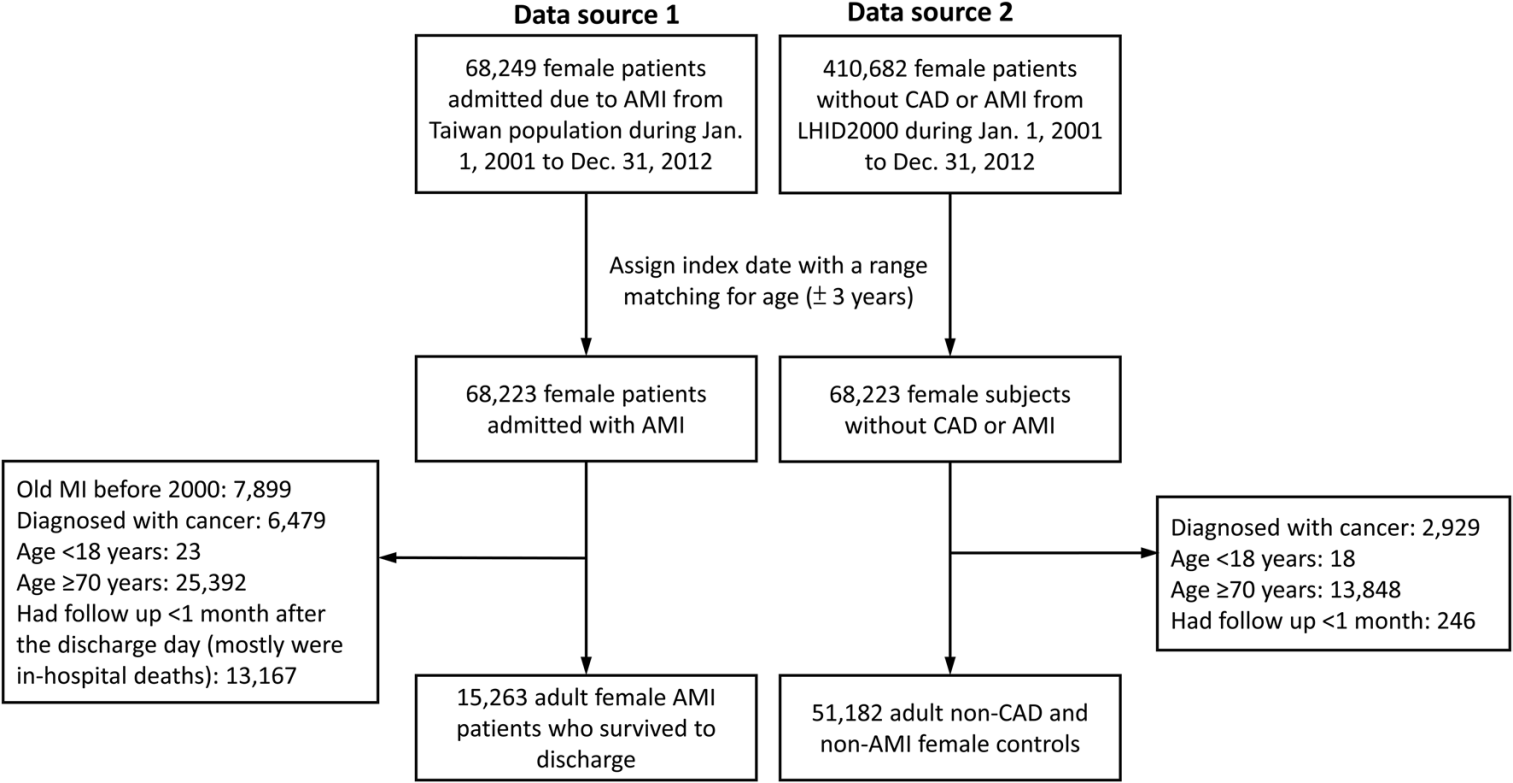
Study design and flow chart of patient inclusion and exclusion. *AMI* acute myocardial infarction, *CAD* coronary artery disease, *LHID2000* Longitudinal Health Insurance Database 2000.

We identified female patients who were admitted to medical facilities with a principal diagnosis of acute MI (*ICD-9-CM* code 410) in the NHIRD between January 1, 2001, and December 31, 2012, as our data source 1 (Fig. 1, left panel). We also identified female patients who had never received a diagnosis of CAD or MI in the LHID2000 as our data source 2 (Fig. 1, right panel). Since individuals with a diagnosis of CAD or MI had been excluded from the LHID2000, the data sources would not capture the same individuals. The accuracy of acute MI diagnoses in the NHIRD was validated previously with a positive predictive value of 0.88, and the percentage of consistency in comorbidity diagnoses was 95.9%^19^.

The index dates of patients with acute MI were defined as the discharge date of the corresponding patient with a history of acute MI and a range matching for age (± 3 years) was performed. The index date of the subject without CAD or MI (the controls) were assigned from the discharge date of the counterpart patient with MI event and a range matching for age (± 3 years) was applied. Patients with a diagnosis of cancer, who were younger than 18 years or older than 70 years were excluded because the World Health Organization recommends organized, population-based breast cancer screening for women aged less than 70 years^20^ and also to reduce the impact of competing deaths. Meanwhile, Taiwan has provided nationwide, free, biennial mammographic screening only for female younger than 70 years^21^. We excluded patients who died during admission for acute MI or soon after discharge, because these patients did not have a chance to develop breast cancer. Information about in-hospital mortality is not provided in the NHIRD; therefore, to exclude the aforementioned patients, we excluded those with a history of acute MI whose follow-up duration was less than 1 month. To avoid the influence of preexisting coronary events, patients who had received a diagnosis of MI before the index date in the acute MI group were also excluded.

### Covariates

Data on baseline covariates were obtained from the relevant claims records with diagnostic codes in the NHIRD and LHID2000 prior to the index date, including age, urbanization level, region of residence, and comorbidities. Urbanization level was used as a proxy for socioeconomic status and was categorized as low, moderate, high, and very high according to population density, the number of college graduates, the number of people older than 65 years, the number of people working in agriculture, and the number of physicians per 100,000 people within each township^22^. The comorbidities included for analysis were hypertension, diabetes mellitus, dyslipidemia, atrial fibrillation, and chronic kidney disease which were strongly associated with MI. A patient was defined as having a given comorbidity if they had at least two outpatient diagnoses or one inpatient diagnosis for that comorbidity within one year before the index date.

### Study outcome

The outcome of the current study was newly diagnosed breast cancer during each patient's follow-up period. In the NHI program, a catastrophic illness certification is issued by the NHI Bureau to patients with major diseases such as stroke, maintenance hemodialysis, or any cancer. Because the diagnosis of breast cancer in the current study was based on the diagnostic code *ICD-9-CM* 174, with the ascertainment of holding a catastrophic illnesses certification, our data should be extremely accurate. The follow-up period was from the index date to the date a diagnosis of breast cancer was received, the date of death, or the end of the study period (December 31, 2012), whichever occurred first.

### Statistical analyses

To adjust for confounding, we conducted inverse probability of treatment weighting (IPTW) with average treatment effect based on propensity score^23^. The propensity score was the predicted probability of having a diagnosis of acute MI given specific covariates (e.g., aged 52 years, with diabetes, and without hypertension) derived from a multivariable logistic regression model without considering the effects of interaction among covariates. All demographics and characteristics, along with the index date (listed in Table 1), were included in the propensity score calculation. The extreme weights by IPTW were common and have great impact on the results, therefore we truncated weights that were above the 99th percentile (without excluding any subjects)^24^. The balance for baseline characteristics between the two groups was evaluated by using standardized difference (STD). An absolute STD value of 0.2 or more represented a substantial difference between groups^25^.

**Table 1:** Demographics and characteristics of patients with AMI and the controls (*N* = 66,445).

Variable	Before IPTW^‡^			After IPTW^‡^		
	AMI (*n* = 15,263)	Non-AMI (*n* = 51,182)	STD	AMI (*n* = 54,952.7)	Non-AMI (*n* = 68,561.2)	STD
Age, year	59.3 ± 8.8	58.7 ± 6.6	0.07	59.2 ± 8.0	59.2 ± 6.9	< 0.01
Age grouping						
18–49 years	2283 (15.0)	3904 (7.6)	0.23	5674.3 (10.3)	5502.9 (8.0)	0.08
50–69 years	12,980 (85.0)	47,278 (92.4)	− 0.23	49,278.5 (89.7)	63,058.3 (92.0)	− 0.08
Urbanization level						
Low	1943 (12.7)	5012 (9.8)	0.09	6020.2 (11.0)	7073. (10.3)	0.02
Moderate	4739 (31.0)	14,854 (29.0)	0.04	16,828.1 (30.6)	20,376.9 (29.7)	0.02
High	4657 (30.5)	15,827 (30.9)	− 0.01	16,904.4 (30.8)	21,125.7 (30.8)	< 0.01
Very high	3924 (25.7)	15,489 (30.3)	− 0.10	15,200 (27.7)	19,985.6 (29.2)	− 0.03
Region						
North	5944 (38.9)	21,594 (42.2)	− 0.07	22,063.5 (40.1)	28,132.8 (41.0)	− 0.02
Central	3479 (22.8)	12,207 (23.9)	− 0.02	12,936.7 (23.5)	16,176.1 (23.6)	< 0.01
South	5158 (33.8)	15,822 (30.9)	0.06	17,861.5 (32.5)	21,948.5 (32.0)	0.01
East	682 (4.5)	1559 (3.0)	0.07	2091.1 (3.8)	2303.9 (3.4)	0.02
Comorbidities						
Hypertension	10,648 (69.8)	10,907 (21.3)	1.11	22,030.1 (40.1)	23,944.6 (34.9)	0.11
Diabetes mellitus	8238 (54.0)	5596 (10.9)	1.04	15,218.3 (27.7)	16,425.1 (24.0)	0.09
Dyslipidemia	6411 (42.0)	5789 (11.3)	0.74	13,385.9 (24.4)	14,432 (21.0)	0.08
Atrial fibrillation	941 (6.17)	121 (0.24)	0.34	1083.2 (2.0)	1156.1 (1.7)	0.02
Chronic kidney disease	4026 (26.4)	1263 (2.5)	0.72	5494.9 (10.0)	6264.2 (9.1)	0.03
Concomitant medications						
All anti-diabetic drugs	7359 (48.2)	5474 (10.7)	0.90	14,207.7 (25.9)	15,388 (22.4)	0.08
Metformin	6466 (42.4)	4583 (9.0)	0.83	12,280.2 (22.3)	13,255.4 (19.3)	0.07
Statin	6008 (39.4)	5932 (11.6)	0.67	13,380.1 (24.3)	14,873.1 (21.7)	0.06
Fibrate	1953 (12.8)	1933 (3.8)	0.33	5147.3 (9.4)	3714.5 (5.4)	0.15
Estrogen use during follow up	340 (2.2)	1716 (3.4)	− 0.07	1589.1 (2.9)	2234.3 (3.3)	− 0.02
Follow-up year	5.2 ± 3.5	6.2 ± 3.2	− 0.31	5.6 ± 3.6	6.0 ± 3.3	− 0.13

*AMI* acute myocardial infarction, *IPTW* inverse probability treatment weighting, *STD* standardized difference.

^‡^Data were presented as frequency (percentage) or mean ± standard deviation.

The incidence of breast cancer was presented using incidence density, which was defined as the number of events per 1000 person-years. The incidence of breast cancer in patients with versus patients without a history of MI was compared using the Cox proportional hazards model. The hazard ratio (HR) and 95% confidence intervals (CI) for the risk of breast cancer among those with an MI compared to those without an MI would be obtained from the Cox model. Furthermore, we conducted a sensitivity analysis to include a lag period to establish a biologically plausible period during which breast cancer could develop following an MI event. To address the unresolved question of whether an MI is associated with breast cancer risk, we established several lag periods, ranging from 6 months to 3 years.

Several pre-specified subgroup analyses were performed by age (dichotomized at 50 years), use of antidiabetic medications and statins, and urbanization level (low and moderate vs high and very high). Because the NHIRD does not contain information on menopause, fifty years of age was chosen as a proxy for menopause as reported in Taiwan previously^26^. A two-sided *P* value of less than 0.05 was considered statistically significant. All analyses were conducted with SAS software (version 9.4, SAS Institute Inc., Cary, NC, USA).

## Results

### Cohort identification

A total of 68,249 female patients who were admitted for acute MI between January 1, 2001, and December 31, 2012, were identified from the NHIRD (data source 1) and 410,682 female patients without a diagnosis of CAD or MI were identified from the LHID2000 database during the same period (data source 2). We identified 68,223 matched female patients without a diagnosis of CAD or MI and 68,223 female patients admitted for acute MI. After exclusion, a total of 66,445 patients were eligible for further analysis, including 15,263 patients with a history of acute MI and 51,182 patients without (Fig. 1).

### Baseline characteristics

Before IPTW, there was no substantial difference in age, urbanization level, or region of residence between patients with a history of acute MI and patients without, with absolute STD values less than 0.2 (Table 1). However, the proportion of premenopausal patients (defined as < 50 years) and the prevalence of all comorbidities were substantially higher in patients with a history of MI than in patients without. The mean follow-up period was also shorter for patients with a history of MI (5.2 years vs 6.2 years, STD =  − 0.31). After IPTW, these covariates were all well balanced (all absolute STD values < 0.2). The mean follow-up period after weighting was 5.6 years for patients with a history of MI and 6.0 years for patients without (STD =  − 0.13).

### Breast cancer after acute MI

**Figure 2:**
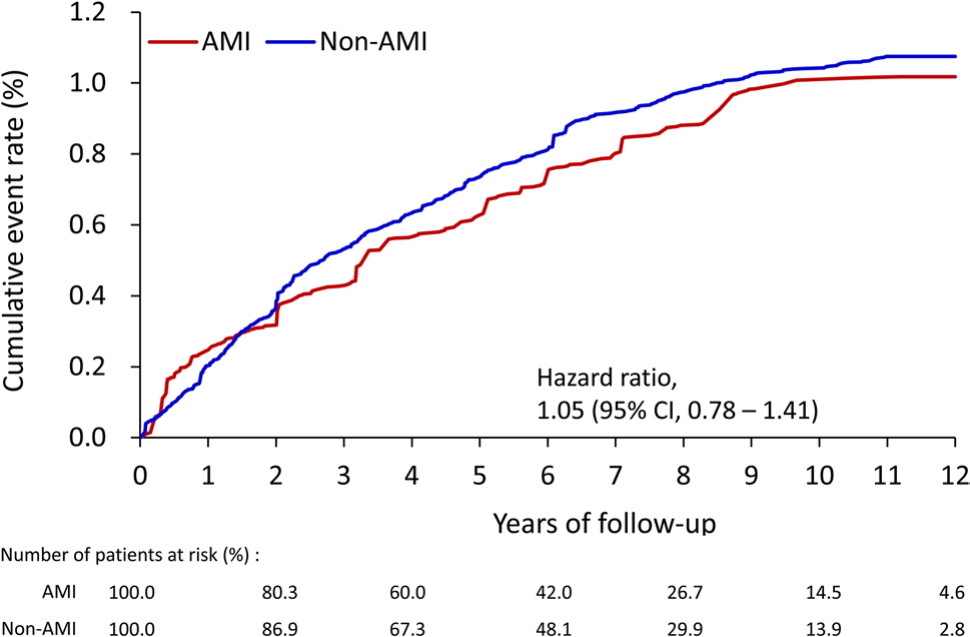
Cumulative event rate of newly diagnosed breast cancer in patients with AMI (red line) and patients without AMI (blue line) in the IPTW-adjusted cohort. *AMI* acute myocardial infarction, *CI* confidence interval, *IPTW* inverse probability of treatment weighting.

Before the IPTW adjustment, 712 patients received a diagnosis of breast cancer during the follow-up period, including 163 patients (1.07%) with a history of acute MI and 549 patients (1.07%) without (HR 1.18, 95% CI 0.98–1.41) (data not shown). After IPTW, the incidence of breast cancer was 1.93 (95% CI 1.78–2.09) and 1.80 (95% CI 1.67–1.93) events per 1,000 person-years among patients with and without a history of MI, respectively, without a significant difference (HR 1.05, 95% CI 0.78–1.41, *P* = 0.756) between the two groups (Fig. 2). The sensitivity analysis including multiple lag periods also disclosed no significant association between MI and breast cancer over times (Table 2).

**Table 2:** Sensitivity analysis for accounting for potential detection bias by adding different lag periods in the IPTW-adjusted cohort.

Lag period	AMI (*n* = 54,952.7)			Non-AMI (*n* = 68,561.2)			HR (95% CI) for AMI	*P* value
	Number of events (%)	Total person years	Incidence (95% CI)*	Number of events (%)	Total person years	Incidence (95% CI)*		
6 months	495.8 (0.9)	304,680.8	1.63 (1.48–1.77)	669.7 (1.0)	410,239.0	1.63 (1.51–1.76)	0.98 (0.71–1.33)	0.875
1 year	453.1 (0.8)	304,680.8	1.49 (1.35–1.62)	598.4 (0.9)	410,239.0	1.46 (1.34–1.58)	1.00 (0.72–1.40)	1.000
2 years	415.2 (0.8)	304,680.8	1.36 (1.23–1.49)	473.7 (0.7)	410,239.0	1.15 (1.05–1.26)	1.16 (0.81–1.67)	0.426
3 years	353.6 (0.6)	304,680.8	1.16 (1.04–1.28)	372.7 (0.5)	410,239.0	0.91 (0.82–1.00)	1.25 (0.83–1.87)	0.282

*IPTW* inverse probability treatment weighting, *AMI* acute myocardial infarction, *CI* confidence interval, *HR* hazard ratio.

*Number of events per 1000 person-years.

**Figure 3:**
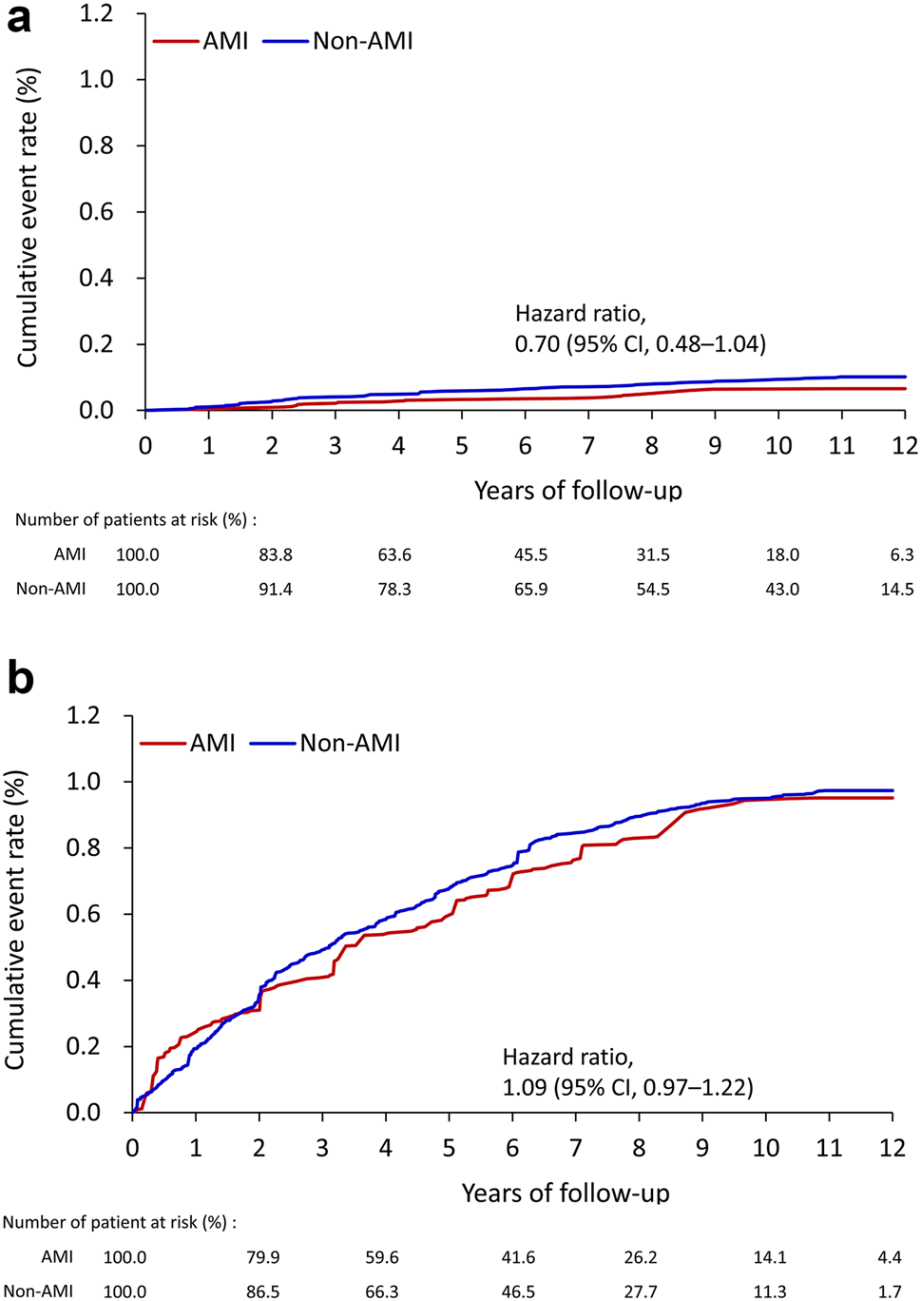
Cumulative event rate of newly diagnosed breast cancer in patients with AMI (red line) and patients without AMI (blue line) in the IPTW-adjusted cohort stratified by age. (**A**) Younger than 50 years, and (**B**) older than 50 years. *AMI* acute myocardial infarction, *CI* confidence interval, *IPTW* inverse probability of treatment weighting.

Subgroup analysis indicated that menopausal status (< 50 years vs ≥ 50 years), use of antidiabetic medications, and urbanization level significantly modified the association between breast cancer and MI status (*P* for interaction < 0.05). Regarding age, the incidence of breast cancer after MI was higher in postmenopausal patients (HR 1.09, 95% CI 0.97–1.22) than in premenopausal patients (HR 0.7, 95% CI 0.48–1.04, *P* for interaction 0.039, Fig. 3A,B). In addition, the association between breast cancer and acute MI was more pronounced in patients who used antidiabetic medications (*P* for interaction 0.041) and who lived in regions with a lower urbanization level (*P* for interaction 0.010, Table 3).

**Table 3:** The association between AMI and the risk of breast cancer in subjects after IPTW adjustment.

Subgroup	AMI (*n* = 54,952.7)			Non-AMI (*n* = 68,561.2)			HR (95% CI)^#^	*P* value
	Number of events (%)	Total person years	Incidence (95% CI)*	Number of events (%)	Total person years	Incidence (95% CI)*		
Total	588.5 (1.1)	304,680.8	1.93 (1.78–2.09)	736.9 (1.1)	410,239.0	1.80 (1.67–1.93)	1.05 (0.78–1.41)	0.756
Age								0.039^‡^
18–50 years	38.6 (0.7)	33,917.9	1.14 (0.78–1.50)	69.5 (1.3)	43,722.9	1.59 (1.22–1.96)	0.70 (0.48–1.04)	
50–69 years	549.9 (1.1)	270,762.9	2.03 (1.86–2.20)	667.4 (1.1)	366,516.1	1.82 (1.68–1.96)	1.09 (0.97–1.22)	
Anti-diabetic drugs								0.041^‡^
No	416.8 (1.0)	243,012.4	1.72 (1.55–1.88)	577.6 (1.1)	334,110.8	1.73 (1.59–1.87)	0.98 (0.86–1.11)	
Yes	171.7 (1.2)	61,668.4	2.78 (2.37–3.20)	159.2 (1.0)	76,128.3	2.09 (1.77–2.42)	1.27 (1.02–1.58)	
Statin user								0.389^‡^
No	429.2 (1.0)	246,055.5	1.74 (1.58–1.91)	573.9 (1.1)	338,651.1	1.69 (1.56–1.83)	1.01 (0.89–1.15)	
Yes	159.3 (1.2)	58,625.3	2.72 (2.29–3.14)	163.0 (1.1)	71,587.9	2.28 (1.93–2.63)	1.13 (0.91–1.41)	
Urbanization level								0.010^‡^
Low/moderate	235.5 (1.0)	127,964.6	1.84 (1.61–2.08)	231.5 (0.8)	163,582.2	1.41 (1.23–1.60)	1.28 (1.06–1.53)	
High/very high	353.0 (1.1)	176,716.2	2.00 (1.79–2.21)	505.4 (1.2)	246,656.9	2.05 (1.87–2.23)	0.95 (0.83–1.09)	

*IPTW* inverse probability treatment weighting, *AMI* acute myocardial infarction, *CI* confidence interval, *HR* hazard ratio.

*Number of events per 1000 person-years.

^#^The comparison of breast cancer risk between patients with AMI versus patients without AMI.

^‡^The P value for interaction.

## Discussion

This study is the first to investigate the association between acute MI and a subsequent diagnosis of breast cancer. We analyzed a large-scale, nationwide cohort comprising more than 66,000 female patients with a follow-up period of 5 years and we found that the risk of breast cancer was not significantly associated with MI status.

The development of cancer is a cumulative, time-dependent process, and the incidence of cancer diagnosis may be influenced by detection bias, which refers to a patient with some diseases receiving a diagnosis for some other diseases due to the increased medical surveillance they are under for the first disease^27^. The cancer detection rate is likely to be higher in the first 6 to 12 months following the diagnosis of other diseases due to detection bias, but the risk may diminish over time when applying a lag period. In the present study, we demonstrated that acute MI does not increase the risk of breast cancer over times by including multiple lag periods in the sensitivity analysis. Although we could not exclude the possibility of detection bias of breast cancer affected by MI, there was no association between the risk of breast cancer and MI status in different lag period even in the first year after the index date in the whole study population. However, we observed a significantly higher incidence of breast cancer following MI among patients residing in less urbanized area and we hypothesized that detection bias might contribute to this trend. Despite Taiwan has provided nationwide and free mammographic screening for female younger than 70 years over the past decades, when compared to patients lived in more urbanized area, patients lived in less urbanized area may undergo more frequent laboratory examinations or breast cancer screening after MI than non-MI patients, which may lead to an increased diagnosis of breast cancer.

In a mouse model of breast cancer, the preexisting tumor burden was significantly increased by coronary artery ligation than sham surgery due to reprogramming of Ly6C^hi^ monocytes in the bone marrow reservoir, which changed them to an immunosuppressive phenotype in both circulation and tumor^16^. Meanwhile, the circulating Ly6C^hi^ monocyte levels and recruitment to tumors were both increased by MI in the mouse model, which was regulated by the innate immune system that drove deleterious cross-disease communication and accelerated tumor growth. In human patients with early-stage breast cancer, the risk of tumor recurrence and cancer-specific death were significantly increased by cardiovascular events (such as MI or stroke) during an average follow-up time of 11.7 years from diagnosis^16^. However, in the present study, we did not find an association between breast cancer and MI status. This may indicate different mechanisms at work in new tumor development compared to existing tumor propagation. Further subclinical studies are required to delineate innate immune reprogramming or other mechanisms at work in the development of breast cancer.

In subgroup analysis of previous studies, acute MI event did not increase the risk of breast cancer^11,13,14,17^. In a Danish study, no increased cancer risk, except for smoking-related cancer, was detected in a group of 96,891 one-year survivors of MI^11^. In patients with ischemic syndromes such as MI and angina pectoris or undergoing revascularization, the incidence of cancer increased for smoking-related cancers, but the incidence of breast cancer in women was similar to the general population (standardized incidence ratio: 0.95, 95% CI: 0.84–1.07)^14^. In another nationwide cohort study including 122,275 patients with a history of MI, when compared to a reference population, the incidence of breast cancer after MI did not increase when adjusted for age, sex, and calendar year (rate ratio 0.87, 95% CI 0.87–1.14)^13^. Furthermore, the same study found that after adjustments for age, sex, calendar year, hypertension, dyslipidemia, diabetes, and socioeconomic status, the incidence of breast cancer was lower among patients who had MI 6 months to 17 years prior to cancer diagnosis than in the reference population (rate ratio 0.82, 95% CI 0.70–0.92). In a largest meta-analysis investigating cancer risk after MI, the incidence of breast cancer in female patients did not increase after MI (odds ratio [OR] 0.94, 95% CI 0.86–1.04)^17^. The aforementioned studies primarily investigated overall rather than focusing specifically on breast cancer risk after MI. The strengths of the present study lie in its specific focus on breast cancer risk and its use of a nationwide dataset. The results are consistent with previous cohort studies that utilized smaller samples. In our study, we observed a lower incidence of breast cancer in patients with a history of MI compared to those without such a history among premenopausal patients when compared to postmenopausal patients, This phenomenon was not observed in other studies and we hypothesize that this discrepancy may stem from other unmeasured confounding factors. Further prospective studies may help to elucidate these associations.

In the present study, we did not calculate the cumulative incidence function using Fine and Gray’s sub-distribution hazard method, which is one of the competing risk survival models, because this approach systematically lowers the incidence of the interested event (e.g., breast cancer) when the proportion of competing risk factors (e.g., death) is higher. In the current study, the long-term mortality rate was inevitably much greater among patients with a history of MI than among those without. The cumulative incidence function using Fine and Gray’s sub-distribution hazard method would be seriously underestimated in patients with a history of MI.

Some extensively used medications, such as antidiabetic medications^28–37^ or statins^38,39^, have been associated with breast cancer. In a Finnish nationwide diabetic study, the use of metformin, other oral antidiabetic medications, and statins was not associated with breast cancer, but a slightly increased risk of breast cancer was observed among insulin users (HR 1.18, 95% CI 1.03–1.36)^28^, which was similar to other studies conducted in Taiwan^32,33^. Another large population-based study conducted in Israel provided evidence of a strong inverse correlation between metformin and breast cancer risk (OR 0.821, 95% CI 0.726–0.928)^29^. Although some population-based studies have concluded that statin usage is associated with a decreased risk of breast cancer^38,39^, two large meta-analyses have shown that statin use was not associated with breast cancer^40,41^. In our study, there was also no association between the use of statins and the incidence of breast cancer both in patients with and without a history of MI. However, among patients who used antidiabetic medications, mainly type 2 diabetes, the risk of breast cancer was higher among patients with than patients without a history of MI. Several studies conducted in Taiwan previously suggested a significantly higher risk of breast cancer among diabetic than non-diabetic patients by using national data^30,37^. Furthermore, the use of different antidiabetic medications may exert different effects on breast cancer risk such as metformin^31^, rosiglitazone^34^ and sitagliptin^35^ were associated with a reduced risk but pioglitazone had a null effect^36^. Therefore, different antidiabetic drugs should be considered separately with regards to their association with the risk of breast cancer. The influence of MI in this population may involve several complex signaling pathways in the diabetes–breast cancer link such as the insulin and insulin-like growth factor family of ligand receptors, dyslipidemia, adipose tissue, and the gut microbiome^42,43^. Further studies investigating the mechanisms tying systemic metabolism to cancer growth are warranted.

### Study limitations

The current analysis was based on large administrative datasets and has several limitations. First, we could not obtain detailed medical and personal data such as dietary habits^44^, smoking habits^45^ or body weight^46^ which are known risk factors for breast cancer and could potentially influence the association between MI and cancer. Additionally, factors like the severity of MI (e.g., Killip classification, revascularization strategies, ejection fraction data) and breast cancer staging could not be estimated in our analysis. Second, we used IPTW to balance the differences between the two study groups. Although all baseline covariates were well balanced after weighting, it did not account for unmeasured confounding factors such as number of healthcare visits. Third, patients with a preexisting MI diagnosis were excluded; thus, these results cannot be extrapolated to patients with a preexisting MI, unstable angina, chronic coronary syndrome, stroke, or other vascular diseases. Fourth, the NHIRD lacks information on menopause status and we used 50 years of age as a proxy, which may not accurately reflect actual menopausal status. Furthermore, breast cancer risk factors can differ between pre- and postmenopausal women of the same age, a distinction we could not fully consider in the present analysis. Finally, the mean follow-up period of 5.6–6.0 years in this study may not have been sufficient to capture all cases of breast cancer development. Further case–control studies with longer follow-up period are necessary to thoroughly investigate the association between a MI event and subsequent breast cancer diagnoses.

## Conclusions

In this study, the incidence of newly diagnosed breast cancer was not increased in patients with a history of MI compared to patients without a history of MI or CAD over a five-year period following the first MI diagnosis.

## Data Availability

The datasets used and analyzed in the current study are available from the corresponding authors upon reasonable request.

## References

[1] Nowbar AN, Howard JP, Finegold JA, Asaria P, Francis DP (2014). global geographic analysis of mortality from ischaemic heart disease by country, age and income: Statistics from World Health Organisation and United Nations. Int. J. Cardiol..

[2] Ralapanawa U, Sivakanesan R (2021). Epidemiology and the magnitude of coronary artery disease and acute coronary syndrome: A narrative review. J. Epidemiol. Glob. Health.

[3] Virani SS (2021). Heart disease and stroke statistics-2021 update: A report from the American Heart Association. Circulation.

[4] Yeh RW (2010). Population trends in the incidence and outcomes of acute myocardial infarction. N. Engl. J. Med..

[5] Chen DY (2020). Cardiovascular toxicity of immune checkpoint inhibitors in cancer patients: A review when cardiology meets immuno-oncology. J. Formos Med. Assoc..

[6] Chen DY (2021). Gonadotropin-releasing hormone antagonist associated with lower cardiovascular risk compared with gonadotropin-releasing hormone agonist in prostate cancer: A nationwide cohort and in vitro study. Prostate.

[7] Chou SH (2023). Sex disparities in the association between acute myocardial infarction and colon cancer risk. Cancer Med..

[8] Meijers WC, de Boer RA (2019). Common risk factors for heart failure and cancer. Cardiovasc. Res..

[9] Sung H (2021). Global cancer statistics 2020: GLOBOCAN estimates of incidence and mortality worldwide for 36 cancers in 185 countries. CA Cancer J. Clin..

[10] Soerjomataram I, Bray F (2021). Planning for tomorrow: Global cancer incidence and the role of prevention 2020–2070. Nat. Rev. Clin. Oncol..

[11] Dreyer L, Olsen JH (1998). Cancer risk of patients discharged with acute myocardial infarct. Epidemiology.

[12] Hasin T (2016). Heart failure after myocardial infarction is associated with increased risk of cancer. J. Am. Coll. Cardiol..

[13] Malmborg M (2018). Incidence of new onset cancer in patients with a myocardial infarction a nationwide cohort study. BMC Cardiovasc. Disord..

[14] Pehrsson SK, Linnersjo A, Hammar N (2005). Cancer risk of patients with ischaemic syndromes. J. Intern. Med..

[15] Rinde LB (2017). Myocardial infarction and future risk of cancer in the general population-the Tromso Study. Eur. J. Epidemiol..

[16] Koelwyn GJ (2020). Myocardial infarction accelerates breast cancer via innate immune reprogramming. Nat. Med..

[17] Li N (2019). Increased cancer risk after myocardial infarction: Fact or fiction? A systemic review and meta-analysis. Cancer Manag. Res..

[18] Lin LY, Warren-Gash C, Smeeth L, Chen PC (2018). Data resource profile: The National Health Insurance Research Database (NHIRD). Epidemiol. Health.

[19] Cheng CL (2014). Validation of acute myocardial infarction cases in the national health insurance research database in taiwan. J. Epidemiol..

[20] *WHO Guidelines Approved by the Guidelines Review Committee* (2014).

[21] Pan HB (2014). The outcome of a quality-controlled mammography screening program: Experience from a population-based study in Taiwan. J. Chin. Med. Assoc..

[22] Liu CY (2006). Incorporating development stratification of Taiwan townships into sampling design of large scale health interview survey. J. Health Manag..

[23] Austin PC, Stuart EA (2015). Moving towards best practice when using inverse probability of treatment weighting (IPTW) using the propensity score to estimate causal treatment effects in observational studies. Stat. Med..

[24] Xiao Y, Moodie EEM, Abrahamowicz M (2013). Comparison of approaches to weight truncation for marginal structural Cox models. Epidemiol. Methods.

[25] McCaffrey DF (2013). A tutorial on propensity score estimation for multiple treatments using generalized boosted models. Stat. Med..

[26] Shen TY, Strong C, Yu T (2020). Age at menopause and mortality in Taiwan: A cohort analysis. Maturitas.

[27] de Jong R (2017). Impact of detection bias on the risk of gastrointestinal cancer and its subsites in type 2 diabetes mellitus. Eur. J. Cancer.

[28] Hosio M (2019). Association of antidiabetic medication and statins with breast cancer incidence in women with type 2 diabetes. Breast Cancer Res. Treat..

[29] Rennert G, Rennert HS, Gronich N, Pinchev M, Gruber SB (2020). Use of metformin and risk of breast and colorectal cancer. Diabetes Res. Clin. Pract..

[30] Tseng CH (2014). Diabetes and breast cancer in Taiwanese women: A detection bias?. Eur. J. Clin. Invest..

[31] Tseng CH (2014). Metformin may reduce breast cancer risk in Taiwanese women with type 2 diabetes. Breast Cancer Res. Treat..

[32] Tseng CH (2015). Use of insulin and mortality from breast cancer among Taiwanese women with diabetes. J. Diabetes Res..

[33] Tseng CH (2015). Prolonged use of human insulin increases breast cancer risk in Taiwanese women with type 2 diabetes. BMC Cancer.

[34] Tseng CH (2017). Rosiglitazone reduces breast cancer risk in Taiwanese female patients with type 2 diabetes mellitus. Oncotarget.

[35] Tseng CH (2017). Sitagliptin may reduce breast cancer risk in women with type 2 diabetes. Clin. Breast Cancer.

[36] Tseng CH (2022). Pioglitazone and breast cancer risk in female patients with type 2 diabetes mellitus: A retrospective cohort analysis. BMC Cancer.

[37] Tseng CH, Chong CK, Tai TY (2009). Secular trend for mortality from breast cancer and the association between diabetes and breast cancer in Taiwan between 1995 and 2006. Diabetologia.

[38] Kim DS, Ahn HS, Kim HJ (2022). Statin use and incidence and mortality of breast and gynecology cancer: A cohort study using the National Health Insurance claims database. Int. J. Cancer.

[39] Marrone MT (2021). Lipid-lowering drug use and cancer incidence and mortality in the ARIC study. JNCI Cancer Spectr..

[40] Islam MM (2017). Exploring association between statin use and breast cancer risk: an updated meta-analysis. Arch. Gynecol. Obstet..

[41] Wu QJ (2015). Statin use and breast cancer survival and risk: A systematic review and meta-analysis. Oncotarget.

[42] Gallagher EJ, LeRoith D (2015). Obesity and diabetes: The increased risk of cancer and cancer-related mortality. Physiol. Rev..

[43] Kang C, LeRoith D, Gallagher EJ (2018). Diabetes, obesity, and breast cancer. Endocrinology.

[44] Tsai HH (2023). The risk of breast cancer between western and Mediterranean dietary patterns. Nutrients.

[45] Cohen SY, Stoll CR, Anandarajah A, Doering M, Colditz GA (2023). Modifiable risk factors in women at high risk of breast cancer: A systematic review. Breast Cancer Res..

[46] Lagarde CB (2024). Obesity-associated epigenetic alterations and the obesity-breast cancer axis. Oncogene.

